# Exercise vs metformin for gestational diabetes mellitus: Protocol for a network meta-analysis

**DOI:** 10.1097/MD.0000000000016038

**Published:** 2019-06-21

**Authors:** Carlos Pascual-Morena, Vicente Martínez-Vizcaíno, Celia Álvarez-Bueno, Diana P. Pozuelo-Carrascosa, Blanca Notario-Pacheco, Alicia Saz-Lara, Rubén Fernández-Rodriguez, Iván Cavero-Redondo

**Affiliations:** a Universidad de Castilla – La Mancha, Health and Social Research Center, Cuenca, Spain; b Universidad Autónoma de Chile, Facultad de Ciencias de la Salud, Talca, Chile.

**Keywords:** diabetes, exercise, gestational diabetes mellitus, metformin, network meta-analysis., physical exercise, pregnancy, protocol

## Abstract

**Introduction:**

The purpose of this protocol is to provide a network meta-analysis methodology that compares the effects of metformin and physical exercise in the prevention and treatment of gestational diabetes mellitus (GDM) and its associated fetal and maternal morbidity.

**Methods and analysis:**

This protocol conforms to the Preferred Reporting Items for Systematic Review and Meta-Analysis Protocols (PRISMA-P) and the recommendations of the Cochrane Collaboration Handbook. An electronic search will be conducted in MEDLINE, EMBASE, Web of Science and the Cochrane Library, from the inception until July 2019. There will be no language restrictions. The Cochrane Collaboration tool for assessing risk of bias (RoB2) and the quality assessment tool for quantitative studies will be used. The Grading of Recommendations, Assessment, Development and Evaluation scale will be used to evaluate the strength of the evidence. A Bayesian network meta-analysis will be carried out, which allows direct and indirect comparison of the interventions, for the risk of GDM, prematurity, caesarean section, macrosomia, hypertensive disorders, insulin requirement, and differences in basal glucose, maternal weight, and weight of the newborn.

**Discussion:**

With this protocol, a methodology is established that resolves the limitations of previous meta-analysis. It will be possible to determine the difference of effect between physical exercise and metformin in the main outcomes of the GDM, as well as the type and intensity of the exercise, and the dose of metformin, more effective.

**Ethics and dissemination:**

The data included in the network meta-analysis will be obtained from trials that meet accepted ethical standards and the Declaration of Helsinki. The results will be published in a peer-reviewed journal. The evidence obtained could be included in the guidelines of clinical practice in pregnancy.

**Strengths and limitations:**

A comprehensive methodology is established for the analysis, through network meta-analysis, of the comparative efficacy of metformin and physical exercise in gestational diabetes mellitus. The results obtained could help medical professionals by establishing the best evidence-based interventions which may be recommended for these population groups.

**Registration number:**

PROSPERO CRD42019121715

## 1 Introduction

Gestational diabetes mellitus (GDM) is defined as glucose intolerance that results in hyperglycemia with onset or first recognition in pregnancy.^[1]^ It is a disorder of varying severity and whose prevalence is about 5.4% in Europe,^[2]^ 7.0% in North America, and 11.2% in South America.^[3]^ GDM increases the risk of short-term complications such as preeclampsia, cesarean delivery, macrosomia, neonatal hypoglycemia, or admission to the neonatal intensive care unit, as well as the long-term development of type 2 diabetes mellitus (T2DM).^[4]^ Some risk factors predispose women to the development of GDM, including overweight or obesity, polycystic ovary syndrome (PCOS), prediabetes, GDM in previous pregnancy, family history of T2DM, advanced maternal age and vitamin D deficiency.^[5]^

GDM management is based on two different approaches: interventions targeted at promoting healthy lifestyles, such as changes in diet or physical activity, and antidiabetic drug treatment.^[6,7]^ Although there is a lack of evidence on the characteristics (i.e., length, frequency, and intensity) of physical activity needed to better manage GDM, the recommendation is to perform moderate aerobic exercise for at least 30 minutes per day.^[8,9]^ When changes in lifestyle fail to control glucose levels, the administration of antidiabetics is recommended. Although insulin was traditionally recommended for gestational diabetes requiring drug treatment, oral antidiabetic agents have recently been considered as potential alternatives, because of their better acceptance, lower cost, and easier administration. Among the alternatives metformin clearly performs better than glibenclamide and has results similar to, or slightly better than, insulin.^[10]^

Numerous systematic reviews and meta-analyses have assessed the effect of metformin or physical exercise versus no intervention in GDM management. However, there is no systematic review or meta-analysis comparing the effect of metformin versus physical exercise in GDM. In addition, no study has compared the effect of different types of exercise on GDM, nor the dose-response effect of different exercise intensities and metformin doses.

### 1.1 Objectives

The protocol of this network meta-analysis provides an objective and replicable method for the extraction and analysis of data to:

1. compare the effect on GDM of metformin and different types of physical exercise; and
2. compare the dose-response effect of different physical exercise intensities and metformin doses.

## 2 Materials and methods

This network meta-analysis protocol has been registered in the PROSPERO database (registration number: CRD42019121715). It will be conducted according to the guidelines of the Preferred Reporting Items for Systematic review and Meta-Analysis Protocols (PRISMA-P)^[11]^ and The Cochrane Handbook for Systematic Reviews of Interventions.^[12]^

### 2.1 Inclusion and exclusion criteria

We will include studies defined by the following characteristics. Type of study: randomized controlled trials (RCT); controlled trials (non-RCT/CT). Type of participant: healthy pregnant women with or without risk factors (i.e., overweight, obesity, PCOS); healthy women with or without risk factors who could get pregnant; pregnant women with established GDM. Type of intervention: treatment with metformin; treatment with supervised or prescribed physical exercise.

We will exclude studies with the following characteristics: studies reporting pre-post analysis without comparison group; physical exercise intervention not specifying type, duration, or frequency; studies combining metformin or physical exercise with other health interventions, such as nutritional interventions, in which data concerning the effect of metformin or physical exercise interventions on GDM cannot be extracted separately.

### 2.2 Outcomes

Main outcomes will include risk of GDM, macrosomia, hypertensive disorders.

Secondary outcomes will include risk of premature birth, caesarean section, and insulin requirement and mean difference of birth weight (gr), weight gain in pregnancy (kg), and fasting plasma glucose (mg/dl).

### 2.3 Search strategy

An electronic search will be carried out in MEDLINE databases (via PubMed), EMBASE, Web of Science, and the Cochrane Library, from inception until July 2019. A second search will be carried out before the final analysis of the data. There will be no language restrictions.

The search strategy for MEDLINE, including the search terms and Boolean operators, is detailed in Table 1. The references in the articles found will be evaluated for inclusion. The same search strategy will be adapted for the other databases.

**Table 1:**
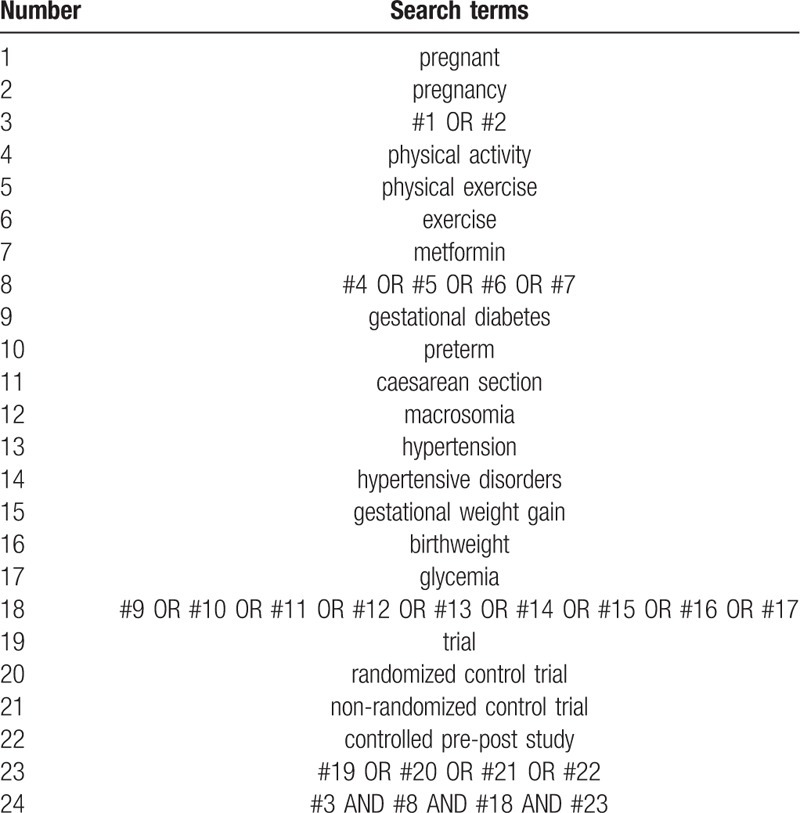
Search strategy for MEDLINE.

### 2.4 Selection and analysis of trials

**Figure 1:**
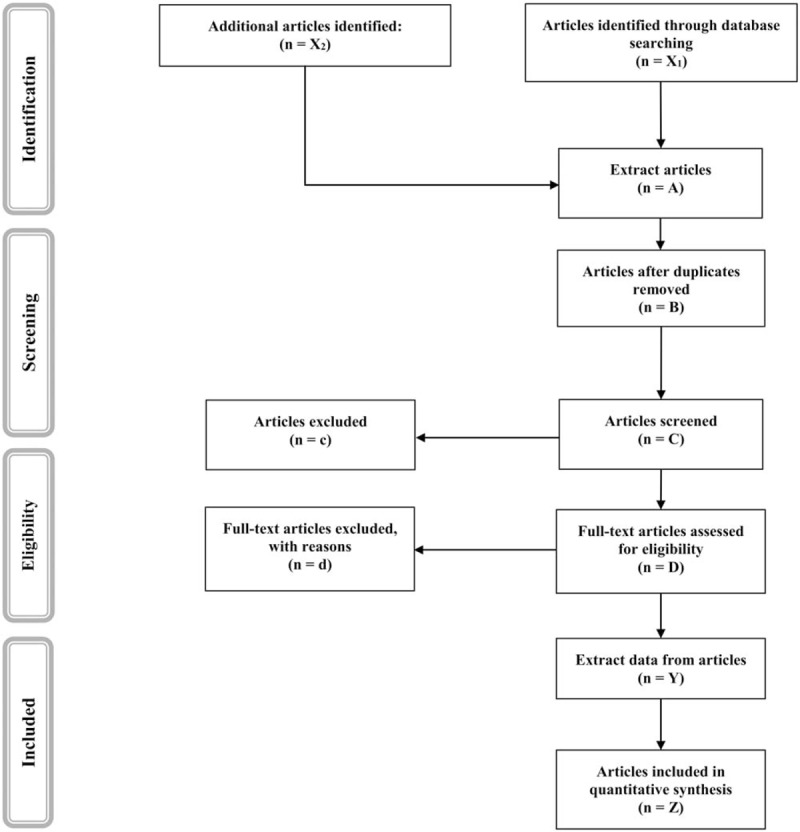
PRISMA flow diagram of identification, screening, eligibility, and inclusion of studies.

After the search is performed, two reviewers will independently screen the titles and abstracts retrieved. The full text of manuscripts selected for inclusion will be examined and the inclusion and exclusion criteria will be applied (Fig. 1). The reviewers will not be blinded to authors, institutions, or journals. Disagreements between reviewers will be resolved by consensus or through the participation of a third reviewer.

The reviewers will independently extract the following information from the included studies: author and year of publication, design of the trial, country, type of intervention (metformin or physical exercise), intervention characteristics (dose, length, setting), population characteristics, number of participants, age of participants, and outcomes studied (Table 2). Any disagreement between reviewers will be resolved by consensus. Finally, study authors will be asked to supply any missing data.

**Table 2:**
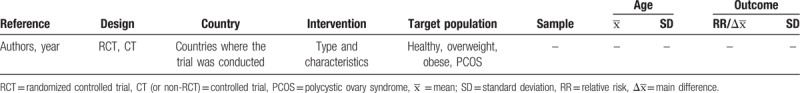
Characteristics of the trials and the sample.

### 2.5 Evaluation of the risk of bias

Two reviewers will independently assess the risk of bias according to the Cochrane Collaboration Handbook recommendations.^[12]^ Disagreements will be resolved by consensus or through the participation of a third reviewer.

The RCTs will be assessed using the Cochrane Collaboration tool for assessing risk of bias (RoB2).^[13]^ This tool assesses the risk of bias according to six domains: bias arising from the randomization process, bias due to deviations from intended interventions, bias due to missing outcome data, bias in measurement of the outcome, bias in selection of the reported result, and overall bias. Overall bias will be considered as “low risk of bias” if the paper have been classified as ‘low risk’ in all domains, “some concerns” if there is at least one domain with rating of ‘some concern’, and “high risk of bias” if there is at least one domain with a ‘high risk’, or several domains with some concerns’ that could affect to the validity of the results.

The Quality Assessment Tool for Quantitative Studies^[14]^ will be used to assess the risk of bias in pre-post studies and non-RCTs. This tool evaluates six domains: selection bias, study design, confounders, blinding, data collection method, withdrawals, and drop-outs. Each domain may be scored as strong, moderate, or weak. The study could be scored as strong, if no domain is qualified as weak, moderate, if only one domain is qualified as weak, or weak, if two or more domains are qualified as weak.

### 2.6 Grading the quality of evidence

The Grading of Recommendations, Assessment, Development and Evaluation (GRADE) tool will be used to assess the quality of the evidence and make recommendations.^[15]^ Each outcome could obtain a high, moderate, low, or very low evidence value, depending on the study design, risk of bias, inconsistency, indirect evidence, imprecision, and publication bias.

### 2.7 Data synthesis

Included clinical trials will be qualitatively summarized in an ad hoc table. The reviewers will determine whether a network meta-analysis is possible after data extraction. A standard meta-analysis for each direct comparison between 2 interventions will be performed using the random effects DerSimonian-Laird method^[16]^ and statistical heterogeneity will be analyzed through calculation of the *I*^*2*^ statistic. Depending on the value of *I*^*2*^, heterogeneity will be considered as not important (0–40%), moderate (30–60%), substantial (50–90%), or considerable (75–100%).^[12]^ In addition, the Egger test will be performed, where *P* <.10 is indicative of heterogeneity.

The values of the main outcomes are dichotomous (incidence or not of the event), summarizing the results in terms of relative risk. For continuous variables, the difference in means will be used. The result will be considered statistically significant when *P* ≤.05. Additionally, the 95% confidence interval (95% CI) will be extracted directly or calculated from the standard deviation, standard error, or in exceptional cases estimated from the *P* value.^[17]^

To compare the dose-response effect of different levels of physical exercise intensity and metformin doses and the effect of different types of physical exercise versus metformin on different outcomes for GDM, a Bayesian network meta-analysis will be performed.^[18]^ We will fit Bayesian models using Markov chain Monte Carlo methods. The model developed by Dias et al will be used for the UK National Institute for Health and Care Excellence Decision Support Unit.^[19]^ The Bucher method will be used to statistically evaluate the consistency of direct and indirect evidence for each analysis.^[20]^. Statistical software STATA 15 (StataCorp) will be used.

Rankograms will be used to show (graphically) the probability that each intervention will be the most effective. Additionally, the surface under the cumulative ranking curve (SUCRA) will be estimated for both interventions. SUCRA has a value of 0 to 1, simplifying the classification of the rankogram, the closer to 1, the more effective the intervention is considered.^[21]^

### 2.8 Analysis by subgroups

Subgroup analysis will be performed based on population characteristics that can modify results for the different outcomes: ’healthy women’, ’overweight or obese women’, and ’women with PCOS’.

## 3 Discussion

The aim of this protocol is to provide a new methodology that overcomes the limitations existing in previous systematic reviews and meta-analyses, which only assess the effect of exercise or the use of metformin separately, without considering the type of exercise or the dose/intensity of the intervention used. With the proposed network meta-analysis, these questions could be answered.

Despite international recommendations stating that diet and physical exercise are the first line option for the prevention and treatment of GDM, antidiabetic drugs are usually used as the first choice treatment, probably due to the low compliance of patients to recommendations on healthy lifestyles and the trend of physicians to medicalize pathological processes.^[22]^ This study is expected to increase the evidence of the effect of physical exercise on GDM, and thus to improve decision making on the part of medical professionals and patients.

Additionally, although physical exercise during pregnancy is recommended (excluding contact sports), there is a lack of evidence on which type of physical exercise is better: aerobic (such as walking, stationary bicycle, or aquatic exercise), resistance or strength, combined, or even alternative therapies such as yoga. There is also no consensus on the most appropriate exercise intensity. Although most guides and authors recommend moderate exercise, there is evidence of a good acceptance of vigorous physical exercise in healthy pregnant women.^[23,24]^

Some concerns should be considered. First, mixed clinical trials, where the effects of physical exercise along with other interventions (dietary) vs control (standard care/advice) are assessed, will likely be found. Only studies separately specifying the effect of each intervention will be included in this network meta-analysis. Second, it is possible that we may find trials where exercise without supervision is prescribed. No special consideration will be made in the analysis, only type and intensity of the prescribed exercise will be taken into account. However, the lack of direct supervision could threaten the validity of the data.

Finally, PCOS is usually treated pharmacologically. When no study reports on physical exercise intervention for the PCOS population, this will be jointly analyzed with the obese population as both have in common an insulin sensitivity alteration.

## 4 Limitations

This network meta-analysis will have as potential limitations those common to systematic reviews, that is, bias due to publication and information. In addition, the non-RCT can be affected by selection bias and allocation concealment, so the homogeneity of the basal characteristics of the intervention and placebo groups are not ensured. To minimize the effect of these limitations, PRISMA-NMA^[25]^ guidelines and the recommendations of the Cochrane Handbook for Systematic Reviews of Interventions^[12]^ will be used.

## Author contributions

**Conceptualization:** Carlos Pascual-Morena, Iván Cavero-Redondo.

**Data curation:** Carlos Pascual-Morena, Vicente Martínez-Vizcaíno.

**Formal analysis:** Carlos Pascual-Morena, Iván Cavero-Redondo.

**Funding acquisition:** Vicente Martínez-Vizcaíno, Blanca Notario-Pacheco.

**Investigation:** Carlos Pascual-Morena.

**Methodology:** Carlos Pascual-Morena, Celia Álvarez-Bueno, Diana P. Pozuelo-Carrascosa, Alicia Saz-Lara, Rubén Fernández-Rodriguez.

**Supervision:** Vicente Martínez-Vizcaíno, Celia Álvarez-Bueno, Iván Cavero-Redondo.

**Writing – original draft:** Vicente Martínez-Vizcaíno, Celia Álvarez-Bueno, Iván Cavero-Redondo.

**Writing – review & editing:** Vicente Martínez-Vizcaíno, Iván Cavero-Redondo.
